# Klf1, a C2H2 Zinc Finger-Transcription Factor, Is Required for Cell Wall Maintenance during Long-Term Quiescence in Differentiated G0 Phase

**DOI:** 10.1371/journal.pone.0078545

**Published:** 2013-10-22

**Authors:** Mizuki Shimanuki, Lisa Uehara, Tomáš Pluskal, Tomoko Yoshida, Aya Kokubu, Yosuke Kawasaki, Mitsuhiro Yanagida

**Affiliations:** G0 Cell Unit, Okinawa Institute of Science and Technology Graduate University (OIST), Onna, Okinawa, Japan; Université Paris-Diderot, France

## Abstract

Fission yeast, Schizoaccharomyces pombe, is a model for studying cellular quiescence. Shifting to a medium that lacks a nitrogen-source induces proliferative cells to enter long-term G0 quiescence. Klf1 is a Krüppel-like transcription factor with a 7-amino acid Cys2His2-type zinc finger motif. The deletion mutant, ∆klf1, normally divides in vegetative medium, but proliferation is not restored after long-term G0 quiescence. Cell biologic, transcriptomic, and metabolomic analyses revealed a unique phenotype of the ∆klf1 mutant in quiescence. Mutant cells had diminished transcripts related to signaling molecules for switching to differentiation; however, proliferative metabolites for cell-wall assembly and antioxidants had significantly increased. Further, the size of ∆klf1 cells increased markedly during quiescence due to the aberrant accumulation of Calcofluor-positive, chitin-like materials beneath the cell wall. After 4 weeks of quiescence, reversible proliferation ability was lost, but metabolism was maintained. Klf1 thus plays a role in G0 phase longevity by enhancing the differentiation signal and suppressing metabolism for growth. If Klf1 is lost, *S. pombe* fails to maintain a constant cell size and normal cell morphology during quiescence.

## Introduction

All cells exist in one of two states, proliferative and quiescent. In the proliferative state, the cell number increases by division, while in the quiescent state, cell life is sustained without division [1,2,3,4,5,6,7]. In multicellular organisms, the majority of cells in tissues and organs are non-dividing, so quiescence is a common cell state. For microbes, quiescence is induced by different environmental conditions, such as nutritional starvation. In the body of a complex organism, some cell types continuously transition between proliferative and quiescent. For example, hematopoietic stem cells give rise to all blood cells during an individual’s lifetime; thus their proliferation and quiescence are carefully regulated [8,9]. The cellular mechanism for the transition between proliferation and quiescence is intriguing.

The fission yeast, *Schizosaccharomyces pombe*, is an excellent model organism for cell and molecular biology studies. This eukaryotic microbe contains a genome of 12 Megabase pairs (Mbp), containing ~5000 protein-coding genes [10,11]. *S. pombe* cells grow rapidly and divide in a defined synthetic medium, EMM2, which contains glucose as the sole carbon source and NH_4_Cl as the sole nitrogen source. Upon removal of NH_4_Cl, S. pombe cells cannot continue to proliferate, but undergo two rounds of cell division in the absence of cell growth, so that cells become small and round and contain pre-replicative DNA. Genome-wide studies of transcripts indicate that a large-scale change in the transcriptome occurs after nitrogen starvation [12,13,14]. Cells enter either a transient G1 phase prior to committing their entry into the sexual phases of mating and meiotic division [15] or the ‘bachelor’ G0 phase, in the absence of cells with the opposite mating type [13,16,17]. That is, fission yeast cells encounter a branch point of cell differentiation after nitrogen source deprivation. They either move toward meiosis, which requires meiotic cell divisions to produce spores, or toward the G0 phase, which requires an asexual, heterothalic cell population. G0-phase cells remain quiescent until replenishment of a nitrogen source [4].

We investigated two types of genes required for G0 quiescence. The first type is called ‘superhousekeeping’ (SHK) genes, as these were initially identified as essential genes by temperature-sensitive (t-s) mutants in the proliferative phase and later also shown to be required for cell quiescence [18,19,20]. The other type, called G0-specific essential, are genes identified by the use of deletion mutants. They proliferate in regular medium, but fail to do so after nitrogen source starvation-induced quiescence [13]. G0-specific genes are of considerable interest relative to cellular systems required for cell survival under starvation. The ∆klf1 deletion mutant loses its ability to restore colony formation when ∆klf1 mutant cells are incubated under nitrogen source (designated -N hereafter) deprivation. G0 medium was later replenished with the nitrogen source, NH_4_Cl.

Previously [13], we reported several G0-specific-essential genes encoding the following proteins: two zinc-finger (designated zf) transcription factors Klf1 and Rsv2; two phosphoinositide-binding proteins involved in autophagy, Atg18 and Mug179/Atg18b; PP2A-like phosphatase inhibitor Sds23/Moc1 and PP2A-like phosphatase activator Ypa1/Rrd1; AMP-kinase regulatory subunit Cbs2; and GTPase-activating proteins Gyp7 and Spac589.07c. In the present study, we investigated the role of Klf1, since its gene deletion mutant showed a unique phenotype in two senses, the late timing of appearance of defects in G0 phase, and the drastically aberrant cell morphology.

## Materials and Methods

### Strains, media, culture, and light microscopy

Basic experimental procedures and media were described previously [13,17]. The *S. pombe* heterothallic haploid 972 h^-^ wild-type cell strain was used. Nitrogen-starved G0 cells were prepared by procedures described previously. Briefly, cells were exponentially grown in EMM2 to a density of 2 x 10^6^ cells/mL at 26°C, harvested by vacuum filtration using a nitrocellulose membrane (0.45 μm pore size), washed in EMM2-N (EMM2 lacking NH_4_Cl) once on the membrane, and then re-suspended in EMM2-N at a concentration of 2 x 10^7^ cells/mL. Because two rounds of cell division rapidly occurred, the resulting concentration reached ~8 x 10^7^ cells/mL. The culture was maintained for 48 h in EMM2-N and used as the source of G0 cells. The nitrogen source was replenished by adding four volumes of fresh EMM2 medium. Cells were harvested at the indicated time points. The deletion mutant ∆klf1 was previously constructed [13]. To establish cells that produce Klf1-GFP or Klf1-FLAG fusion protein (tagged at the C-terminus of Klf1), fused genes encoding either Klf1-GFP or Klf1-FLAG were integrated into the chromosome at the native klf1 locus of wild-type 972 h^-^ cells, together with the kan^r^ gene as a screening marker. DNA fragments containing the klf1-GFP fusion gene or the klf1-3xFLAG-tag, and the kan^r^ marker gene, were constructed by polymerase chain reaction using the pFA6a-GFPS65T-kanMX6 plasmid and pFA6a-3xFLAG-kanMX6 plasmid, respectively, as a template. Fluorescence microscopy was performed using a DeltaVision microscope system (Applied Precision Inc.) with deconvolution technology to reduce the noise.

### Immunoblotting and immunoprecipitation

Immunoblot procedures were described previously [13]. Anti-GFP antibody (Sigma Chemical Co., St. Louis, MO), anti-FLAG antibody (Sigma) and anti-α-tubulin (a gift from K. Gull) were used as primary antibodies. Horseradish peroxidase-conjugated secondary antibodies and an ECL chemiluminescence system (Amersham, Piscataway, NJ) were used to amplify signal expression. Cell extracts were prepared as described in Hayashi et al. [21]. Briefly, exponentially growing cells (1 x10^11^) carrying chromosomally integrated klf1-GFP or klf1-FLAG expressed under the native promoter were lysed in extraction buffer (25 mM HEPES–KOH pH 7.5, 200 mM NaCl, 10% glycerol, 0.1% NP-40, 1 mM phenylmethylsulfonyl fluoride) supplemented with a protease inhibitor cocktail (Sigma). Extracts were centrifuged twice (20 min at 7600 rpm and 30 min at 20,000 rpm), and incubated with anti-FLAG M2 affinity gel (Sigma) for 2 h. Beads were then washed with extraction buffer. Eluates were obtained by incubating with 150 μg/mL 3x FLAG peptide (Sigma).

### Electron microscopy

Transmission electron microscopy procedures were described previously [18]. Cells were fixed with 2% glutaraldehyde in 100 mM phosphate buffer pH 7.2 for 2 h at 26°C, post-fixed with 2% potassium permanganate overnight at 4°C, and embedded in Epon812 (TAAB). Ultra-thin sections were stained in 2% uranyl acetate and Reynold’s lead citrate, and viewed with a TEM JEM1230R (JEOL) operating at 100 kV.

### Transcriptomic analysis

Transcriptomic data were acquired by DNA microarray hybridization using the Yeast Genome 2.0 GeneChip (Affymetrix). Procedures used for DNA microarray hybridization were described previously [18]. Data were normalized using MAS 5.0. Procedures for clustering gene expression profiles were basically the same as described previously [13,18]. SPEXS software version 0.17 [22] was used to discover sequence motifs in the 800-bp upstream regions of the genes. Gene Ontology data and genome sequence data used for this analysis were retrieved from the Nov 28, 2006 version of the GeneDB database (now superseded by PomBase [23]).

### Transcriptome data accession number

Raw microarray data, metadata and resulting scores were deposited into the NCBI GEO (Gene Expression Omnibus) Database (URL: http://www.ncbi.nlm.nih.gov/geo/) under the accession number GSE50986.

### Proteomic analysis

Immunopurified samples were separated with sodium dodecyl sulfate-polyacrylamide gel electrophoresis (SDS-PAGE), 12.5% acrylamide (including 0.5% N,N’-methylenebisacrylamide), and visualized with Coomassie Brilliant Blue staining. The area from the top to the bottom of the separation gel was cut at ~1 to 2-mm intervals. After in-gel digestion with modified trypsin (Roche, Nutley, NJ), peptides were analyzed by online liquid chromatography-tandem mass spectrometry on an LTQ Orbitrap (Thermo Fisher). Other procedures and data analyses were performed essentially as described previously [21,24,25].

### Metabolomic analysis

The *S. pombe* metabolome analysis was performed as previously described [26,27]. Briefly, cells from cultures (50 mL/sample, 3.3 × 10^6^ cells/mL for vegetative cells, or 10^7^ cells/mLfor G0 cells, respectively) were collected by vacuum filtration and immediately quenched in 25 mL of -40°C methanol. Three times more G0 cells than VE cells were used in order to adjust the amount of total cell protein, since the cell size in G0 is approximately one third of the average cell size in VE. Cells were harvested by centrifugation and internal standards (10 nmol of HEPES and PIPES) were added to each sample. Cells were disrupted using a Multi-Beads Shocker (Yasui Kikai). Proteins were removed by filtering on an Amicon Ultra 10-kDa cut-off filter (Millipore) and samples were concentrated by vacuum evaporation. Finally, each sample was resuspended in 40 µL of 50% acetonitrile and 1 µL was used per MS injection. Raw mass spectrometry (MS) data were analyzed using the MZmine 2 software [28]. Retention times of metabolites reported in this manuscript were verified by analyzing pure standards, with the exception of N-acetyl-D-glucosaminate, which was identified based on the chemical formula prediction from the MS data [29].

## Results

### GFP-fused Klf1, a Krüppel-like factor in *S. pombe*, shifts its Intracellular localization to chromatin in the G0 phase

**Figure 1:**
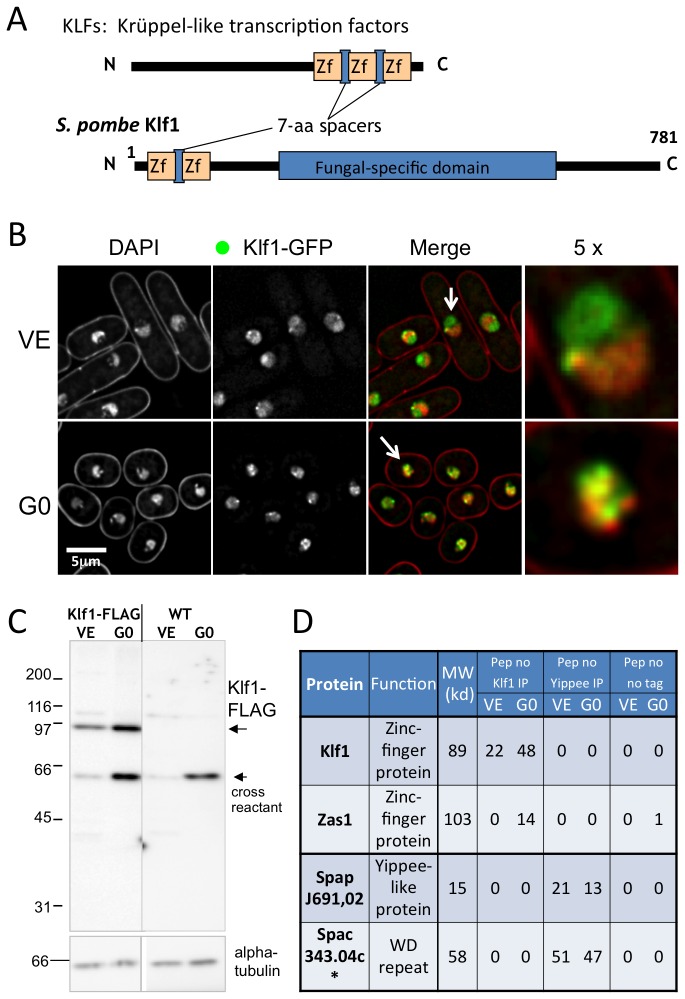
In G0 phase, Klf1, a C2H2 zinc finger transcription factor, localizes in nuclear chromatin, increases in amount, and co-precipitates with Zas1. A. *S. pombe* Klf1 contains two C2H2 zinc finger motifs spaced by seven amino acids, resembling Krüppel-like transcription factors. The central to carboxy-terminal domains contain a sequence also found in *S. pombe* Zas1 and *S. cerevisiae* YPR02c. B. The klf1^+^ gene tagged with GFP was chromosomally integrated and expressed under the native promoter in wild-type (WT) vegetative (VE) and G0 cells. DAPI stains DNA. Merged images of Klf1-GFP (green) and DNA (red) are also shown. The 5-fold enlarged merged images of nuclei indicated by arrows show that Klf1 in VE cells locates in the non-chromatin nucleolar region, while Klf1 in G0 cells locates preferentially in the chromatin region. Punctate localization of Klf1 is also observed. C. Immunoblotting was performed to detect Klf1-FLAG protein expressed in VE and G0 cells. As a control, extracts of WT cells not containing the chromosomally integrated Klf1-FLAG were used. The loading control was α-tubulin detected by antibody TAT1 (Materials and Methods). Klf1-FLAG levels were higher in G0 cells than in VE cells. D. Mass spectrometric analysis of immunoprecipitates obtained with antibody against FLAG for the extracts of cells that expressed the chromosomally integrated Klf1-FLAG (see text). Proteins were run in SDS-PAGE, extracted, and digested with trypsin prior to LC/MS analysis. The molecular weight and number of peptides detected are shown. As a control, the chromosomally integrated gene Ype1/Spapj691.02 (Yippee-like protein, see text) was tagged with FLAG and expressed under the native promoter, and immunoprecipitated with an antibody against FLAG. In the extracts of VE cells, Klf1-FLAG precipitated alone, whereas in the G0 extracts, Klf1-FLAG co-precipitated with Zas1 protein, which is also a zinc-finger protein similar to Klf1 (see text). Control Ype1/Spapj691.02 protein was co-precipitated with another protein, Gid7/Spac343.04c, in VE and also in G0. These data indicate that the stress responsive Ype1/ Spapj691.02 forms a complex with Gid7/ Spac343.04c, which contains a WD repeat and is implicated in pheromone-dependent signal transduction [51]. There is thus no cross-precipitation between Klf1-FLAG and Ype1/Spapj691.02-FLAG. Other control extracts used were obtained from no-tag cells, and results of no-tag proteins precipitated by antibody against FLAG are also shown.

The *S. pombe* klf1^+^ gene encodes a 781-amino acid protein (predicted MW 89.0 kDa), containing two Cys_2_His_2_ (C2H2) type zinc finger motifs in the amino terminal domain. C2H2 motifs are spaced by 7 amino acids (Figure 1A). In this regard, Klf1 is similar to higher eukaryotic KLFs, Krüppel-like transcription factors ([30]; See Discussion).

The Klf1 protein was tagged with green fluorescence protein (GFP) at the carboxy-terminus, encoded by the fusion gene chromosomally integrated at the native klf1 locus, and expressed under the native promoter (Materials and Methods). The integrant strain was checked for colony-forming ability after a 4-week incubation under nitrogen starvation in G0 phase, and no significant defect was found. Rod-shaped cells grown under vegetative division (VE) were observed by fluorescence microscopy (Figure 1B, top panel). In the merged image, green and red staining represents Klf1-GFP and chromatin, respectively. Comparison with the 4',6-diamidino-2-phenylindole (DAPI) staining pattern revealed that KLF1-GFP accumulated in the nucleolar region [31,32] and not in the chromatin region in vegetative cells (see also the enlarged photograph of nucleus in the rightmost panel of Figure 1B, showing the separation of DNA and Klf1-GFP). In contrast, G0-phase round cells produced under N-starvation accumulated Klf1-GFP signals in the nuclear chromatin region stained with DAPI (G0, right panel). In Figure S1, signals observed in the green channel of non-integrant wild-type cells were scarce, indicating that auto-fluorescence was negligible in the Klf1-GFP images under our experimental conditions. Taken together, these findings indicate that Klf1-GFP is localized in the nucleus, preferentially in the nucleolar and chromatin regions in the VE and G0 phases, respectively. Klf1 seems to mobilize to the chromatin region during G0. Punctate localization of Klf1-GFP was observed in both VE and G0 cells. The identity of this punctate nuclear signal is unknown. Dots are often observed along the nuclear envelope, possibly related to peri-telomeric regions [33]..

### Klf1 protein level increases in G0

We next attempted to detect Klf1 protein using the fusion gene integrant strain which produces Klf1-3xFLAG-tag fusion protein instead of the inherent Klf1. The integrant strain was checked for colony formation ability after a 4-week incubation under nitrogen starvation in G0 phase, and no significant defect was found. VE and G0 cell extracts were prepared and run in SDS-PAGE (Materials and Methods). Klf1-FLAG protein was detected by an antibody against FLAG-tag at the position of the predicted MW, using tubulin as the loading control, which was detected by the antibody TAT1 (Materials and Methods). No band was detected at the corresponding MW position in the control of non-tagged wild-type cell extracts. Based on the band intensity (Figure 1C), the Klf1-FLAG protein level was significantly increased (about 3-fold) in N-starved G0 cells in comparison with VE phase. The equivalent level of increase was also detected with Klf1-GFP protein (Figure S1B). It is consistent with the results from DNA microarray experiment that klf1^+^ transcripts increased 3-4-fold more in G0 than in VE.

### Another zf protein co-precipitates with Klf1-FLAG in G0

To identify protein(s) that were bound to Klf1-FLAG in cells of VE or G0 phase, immunoprecipitation was performed using the constructed Klf1-FLAG integrant strains and beads containing antibodies against FLAG (control immunoblot data shown in Figure 1C). Note that the level of Klf1-FLAG was increased in G0 phase. Mass spectrometry was used to analyze proteins precipitated according to previously described procedures [21,24]. As a control for the specificity of immunopreciptated proteins, Yippee-like protein (SpapJ691.02, designated Ype1 hereafter) tagged with FLAG, which is chromosomally integrated and expressed under the native promoter, was similarly analyzed. Ype1, like Klf1, is non-essential in VE phase, but becomes essential for G0 maintenance or exit from G0 [13].

Control Ype1 co-precipitated with Spac343.04c (designated Gid7 hereafter as it is similar to *S. cerevisiae* Gid7) in both the VE and G0 phase, but not at all with Klf1 (Figure 1D). On the other hand, Klf1-FLAG did not precipitate with any other protein in VE phase, but did co-precipitate with Zas1, suggesting that Zas1 interacts with Klf1 only in G0. Zas1 is another Krüppel-like transcription factor in *S. pombe* that is essential in VE phase (see Discussion). Transcription of zas1^+^ does not increase in G0, however, in contrast with that of klf1^+^.

### Cell size of the ∆klf1 mutant increases in concert with aberrant cell wall morphology during long-term incubation in G0

**Figure 2:**
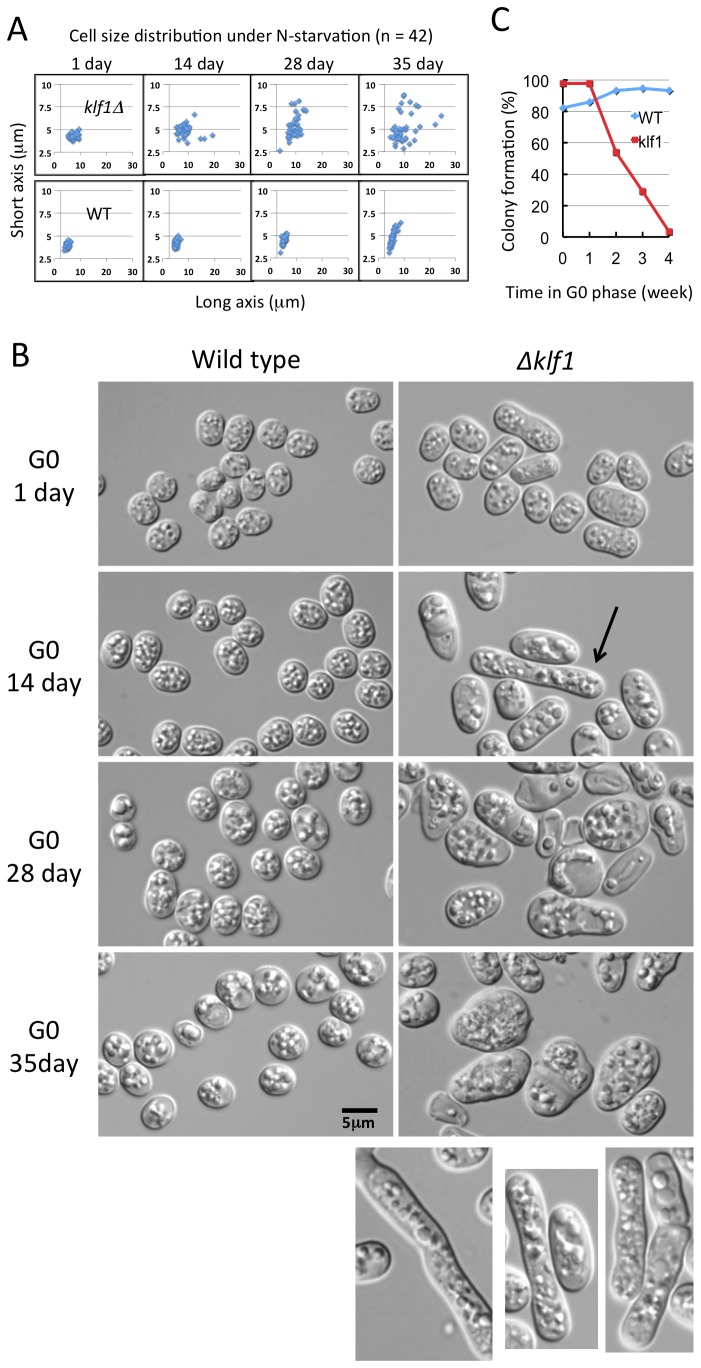
∆klf1 mutant cells in G0 phase exhibit increased volume. A. Cell sizes (long and short axes) of individual wild-type and ∆klf1 deletion mutant G0 cells were measured (Table S1). ∆klf1 cells in G0 at 1 d were somewhat asymmetric and larger than wild-type cells. After 14, 28, and 35 d, mutant cells showed progressively larger volumes, whereas wild-type cells maintained approximately the same size for 28 d. B. Wild-type and mutant ∆klf1 cells were observed on G0 after 1, 14, 28, and 35 d. Mutant ∆klf1 cells were already bigger than WT in G0 at 1 d, and their volume increased further during the next 14 d. Approximately 2% of mutant cells were significantly elongated and rod-like after 14 d (indicated by the arrow and also shown in the bottom right). Such rod-like cells were not observed among wild-type cells in G0. C. Wild-type and ∆klf1 mutant cells were cultured in G0 for four weeks. Cells were plated on complete EMM2 medium to assay cells capable of restoring colony formation. After one week in G0, percent colony formation of the ∆klf1 mutant was nearly 100%, whereas it decreased to 55%, 30%, and 5% after two, three, and four weeks in G0, respectively.

The rod-like shape and size of ∆klf1 mutant cells in VE medium are indistinguishable from those of wild type cells. ∆klf1 cells change into round in G0 phase, as wild type. However, their cell size increased and morphology became abnormal under long term N-starvation (quantitative data shown in Figure 2A and Table S1, and micrographs in Figure 2B). The cell size distribution of WT and ∆klf1 mutant cells was measured at 1, 14, 28, and 35 days (d). Mutant cell size (7.36 µm, on average, long axis; range, 5.34-9.57 µm) was already larger than WT (5.22 µm; 3.86-6.49 µm, do.) cell size at 1 d and increased further after two to five weeks (10.13 µm; 4.84-24.51 µm, do.). Although a slight increase of cell size and size range was observed in WT after five weeks (5.66 µm; 4.25-8.75 µm, do.), ∆klf1 mutant cells showed not only a greater increase in size, but also deformed cell shape (Figure 2B). Klf1 thus seems to be required for maintenance of uniform cell size and morphology in G0. 

We then measured cell viability after 1, 2, 3, and 4 weeks in the G0 phase. After 4 weeks, ∆klf1 mutant cells lost the ability to form colonies upon being shifted to vegetative culture medium (Figure 2C). The high viability of mutant cells was still maintained after 1 week, but it was considerably lower (~50%) after 2 weeks. Only 1% to 2% of the cells maintained their ability to restore colony formation after 4 weeks. In contrast, wild-type cells maintained high viability for more than 4 weeks. These results confirmed the previous results [13].

Aberrantly-shaped large ∆klf1 cells were highly abundant after 2 to 5 weeks in G0. Mutant cells were stained by calcofluor-white [34], which stains chitin, a long-chain polymer of (β-1,4 linked) N-acetyl glucosamine (Calcofluor also stains cellulose, a polymer of β-1,4 linked glucose, in plants) (Figure S2). A lump-like deposition of brightly-stained material was observed along the inner side of the mutant cell wall in G0 phase after 2 weeks, but not in wild-type G0 cells. These results suggested that ∆klf1 cells had severely impaired cell wall organization. Aberrant deposition of chitin-like materials was not observed, however, in ∆klf1 mutant cells in the vegetative phase, or after at least 1 day in G0. Calcofluor was highly positive to the newly made cell wall, of which N-acetyl-D-glucosamine is a major component.

### Cell division of ∆klf1 mutant is not restored upon nutritional replenishment

**Figure 3:**
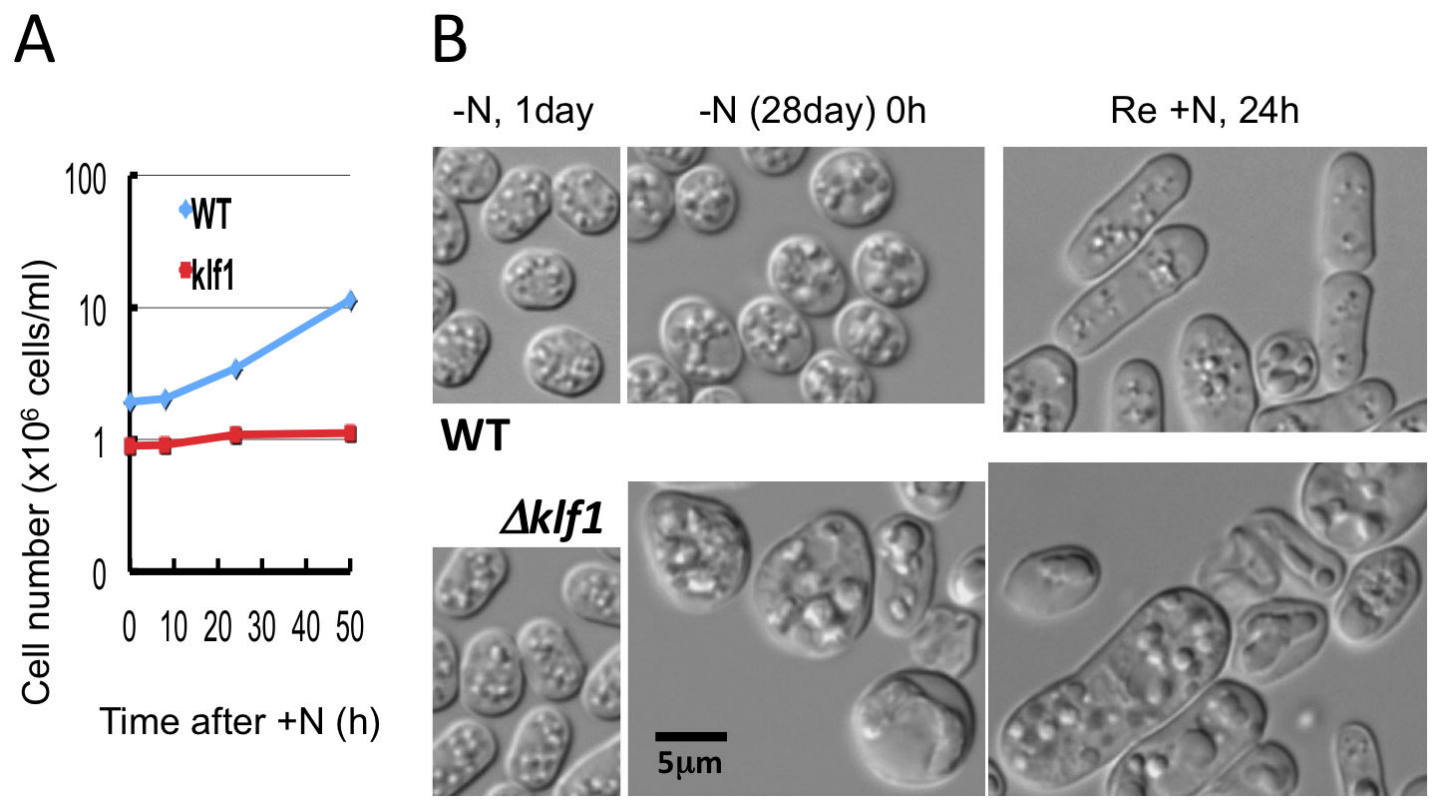
Cell division of ∆klf1 mutant cells maintained in G0 phase for 28 d was not restored in replenished medium. A. Wild-type (WT) and ∆klf1 mutant cells were cultured in G0 phase (-N) for 28 d, and then shifted back to medium containing nitrogen (+N). The cell number was then counted for 0 to 50 h. The number of WT cells (blue line) began to increase after 10 h, whereas the number of mutant cells (red line) failed to increase. B. WT and mutant cells in the same culture as described above were observed under a light microscope. WT cells became rod-shaped after 24 h in the presence of a nitrogen source, but the ∆klf1 mutant cells remained large and swelled, assuming a deformed shape.

We then examined whether ∆klf1 cells in G0 could divide once or twice upon nutritional replenishment, despite their failure to produce colonies. WT and ∆klf1 cells were thus incubated in G0 phase for 28 d and then shifted to the complete synthetic medium (EMM2), and the cell number was measured. WT cells began to divide after 10 h, and the cell number increased ~5-fold after 50 h (Figure 3A). The number of ∆klf1 mutant cells, however, did not increase at all.

WT cells were initially small and round before nutritional replenishment (Figure 3B, -N 28 d, 0 h), and many of them became rod-shaped vegetative cells (Re +N, 24h, top panel). Mutant ∆klf1 cells, on the other hand, remained aberrantly shaped (Re +N, 24h, bottom). These findings indicated that the growth and division of ∆klf1 mutant cells did not respond to nutritional replenishment, but based on the change in cell shape some metabolic changes occurred.

### After nitrogen starvation ∆klf1 cells show increased cell volume and aberrant morphology

**Figure 4:**
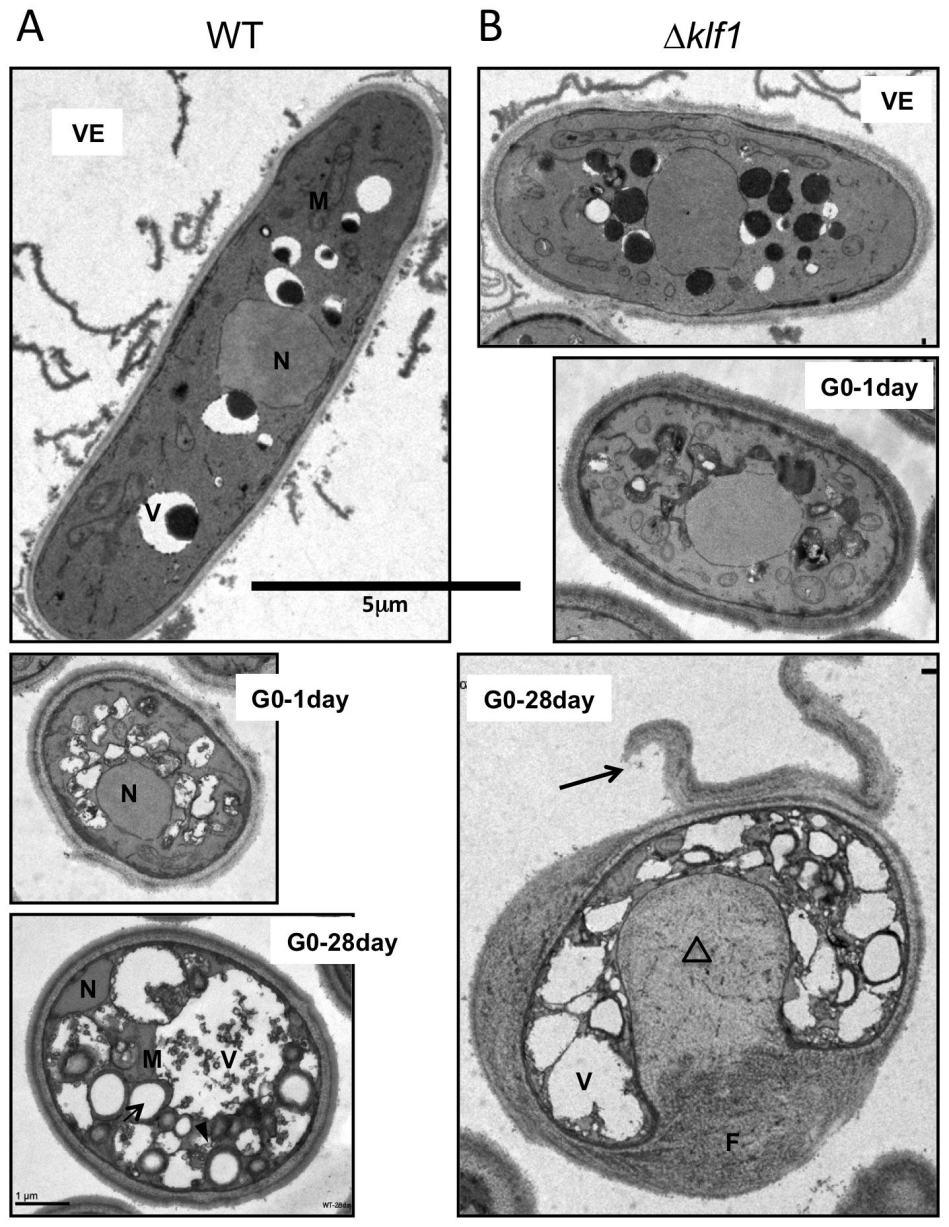
Thin-section electron micrographs of WT and ∆klf1 cells in G0 phase. The glutaraldehyde and KMnO_4_ method was applied to prepare specimens for electron microscopy (Materials and Methods). Wild-type and ∆klf1 cultures in VE medium and G0 phase for 1 and 28 d were observed. A. Wild-type (WT) cells; vegetatively grown (top), G0 phase (1 d, middle and 28 d, bottom). N, nucleus; V, vacuole; M, mitochondria; arrow and arrowhead, unidentified organelle (see text). B. ∆klf1 mutant cells. vegetatively grown (top), G0 phase (1 d middle and 28 d bottom). V, vacuole; F, a fibrous layer-like structure (see text); arrow, peeled-off cell wall; triangle, structures protruding toward the cytoplasm. Bar, 5 µm.

WT and ∆klf1 mutant cells in VE were rod-shaped, with a centrally located nucleus and various cellular organelles (Figure 4A and B, top). At 1 d (90-100% viable) in G0 after the nutritional shift, ∆klf1 cells were bigger than WT cells, despite their similar appearance (Figure 4A and B, middle).

**Figure 5:**
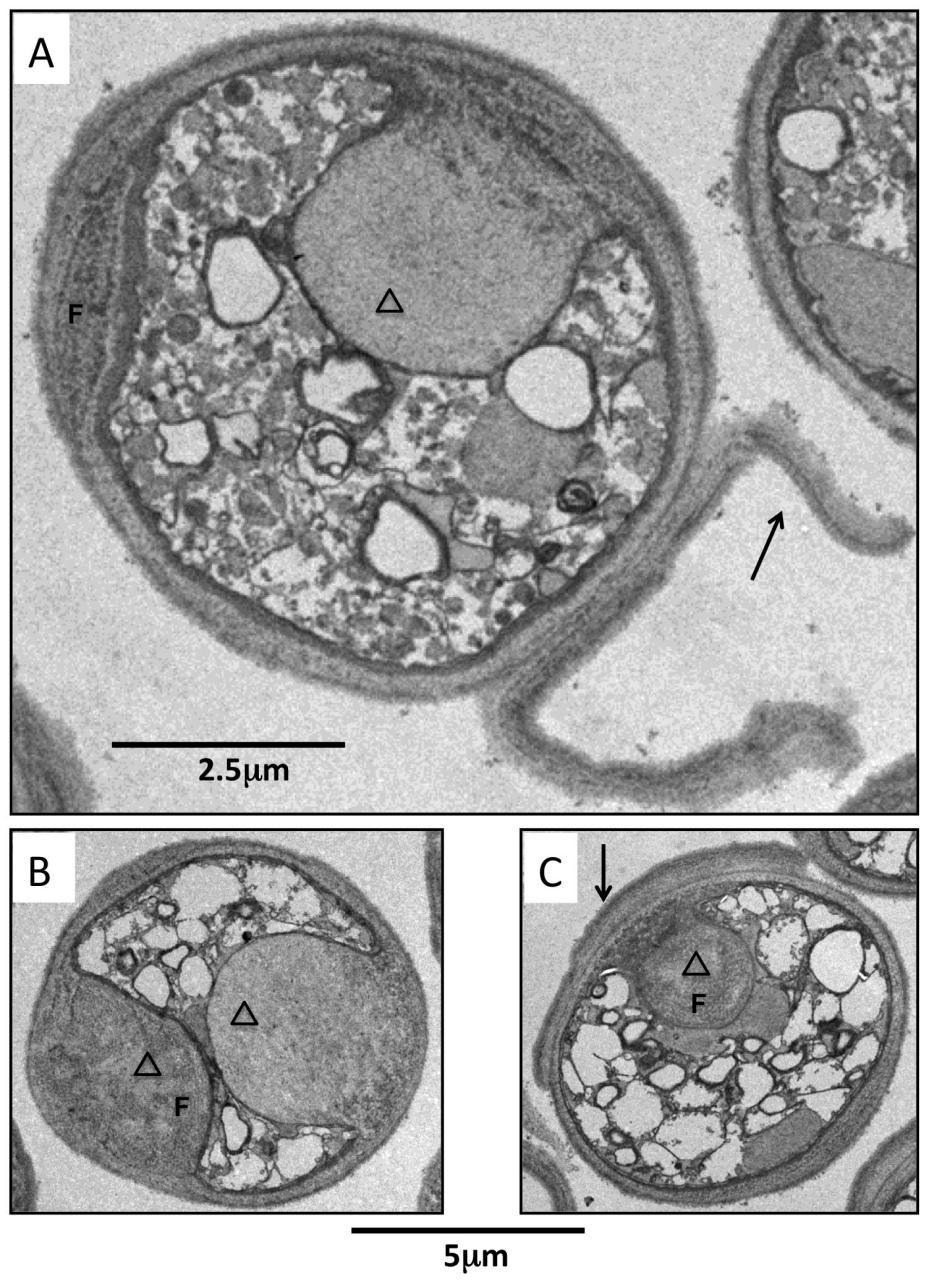
Three electron micrographs of ∆klf1 mutant cells in G0 phase after 28 d. A-C. In all mutant cells, cell wall organization and cytoplasm were abnormal. F denotes a fibrous layer-like structure (see text); arrow, peeled-off cell wall; triangle, structures protruding toward the cytoplasm. Scale bar on A is 2.5 µm; on B and C, 5 µm.

After 28 d in G0 phase, the appearance of WT and ∆klf1 cells differed greatly (Figure 4A and B, bottom). The cell envelope and intracellular architecture of ∆klf1 were remarkably disorganized. Vacuole-like organelles were crammed into the cytoplasm. The cell wall was often peeled off in ∆klf1 mutant cells (Figure 4B, bottom, Figure 5A and C). Certain materials, possibly wall components, appeared to accumulate between the cell surface and the cytoplasmic boundary. Layer-like or fibrous networks were observed in the accumulated material, which seemed to cause the volume increase of ∆klf1 mutant cells. The accumulated materials protruded toward the interior of the cell, pushing the cytoplasm aside, sometimes at multiple sites (Figure 5A and B). We speculate that the materials, possibly the cell wall precursors, accumulated beneath the cell surface, and some became new cell wall, so that partly peeled-off wall might be frequently observed in ∆klf1 mutant cells. The cell size of ∆klf1 mutant cells was thus increased, probably due to the aberrant accumulation of materials or the addition of newly made cell walls.

Possibly relevant to the senescence of G0 cells under long-term N-starvation, even WT cells in G0 after 4 weeks looked rather different from those after only 1 day: larger and highly abundant vacuoles, autophagy sites, unknown organelles appeared as large and small spherical structures, translocated nucleus to peripheral area. However, cell wall structure at 4 weeks in the WT cells was indistinguishable from that at 1 day, in sharp contrast to that of ∆klf1 mutant cells.

### Vegetative transcriptome of ∆klf1 is similar to that of WT

To gain insight into the role of the Klf1 transcription factor, we performed a genome-wide transcriptomic analysis to compare the levels of individual transcripts between WT and ∆klf1 mutant cells in VE and G0 phases. Procedures for DNA microarray hybridization using Affimetrix Genechip Yeast Array are described in the Materials and Methods. Both WT and ∆klf1 mutant strain cells were brought to the nitrogen-starved G0 phase at 26°C for 24 h. Two identical sets of experiments were performed. Detailed transcriptomic data are presented in Supporting Information (Table S2).

The transcriptomic difference between ∆klf1 and WT cells in the VE phase was rather small. Only one transcript (spac5H10.01, encoding a protein containing a motif DUF1445 of unknown function) was reproducibly diminished below 1/6-fold in ∆klf1 cells in VE. The physiologic relevance of this decrease is unknown. A small (~1/2) but reproducible decrease of two other transcripts, spac11D3.18c and isp5, was observed in ∆klf1 VE cells. These two transcripts are presumed to encode the plasma membrane transporters for nicotinamide mononucleotide and amino acids, respectively. Otherwise the transcriptomic patterns of WT and ∆klf1 in the VE phase were indistinguishable.

### Thirteen transcripts show comparative changes in ∆klf1 G0 cells

**Figure 6:**
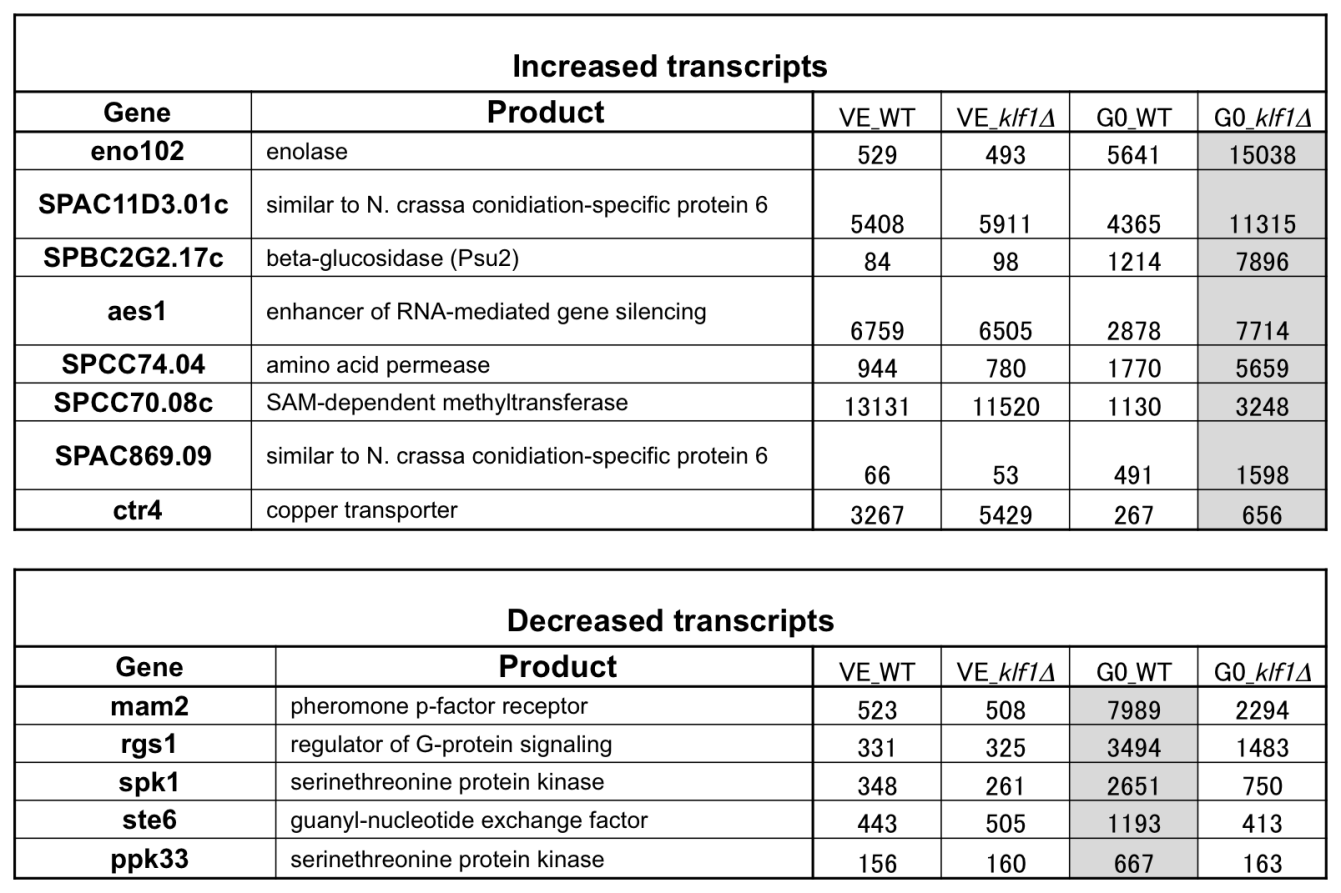
Transcriptomic comparison between wild-type and ∆klf1 mutant cells in the VE or G0 phase. Eight transcripts that increased and five that decreased in ∆klf1 G0 cells are shown with annotated gene functions and measured transcript intensity (see text).

Levels of 13 transcripts were reproducibly altered (8 increased, 5 decreased) in ∆klf1 cells (G0) compared with WT cells in nitrogen-deficient medium at 26°C for 24 h (Figure 6). Genes that decreased were mam2^+^, ste6^+^, rgs1^+^, spk1^+^, and ppk33^+^. These genes are all required for differentiation and sexual development in *S. pombe* [35,36,37,38]. In WT cells in VE, the transcript levels of these genes were relatively low, but all increased greatly under nitrogen starvation. This increase, however, was significantly restrained in ∆klf1 mutant cells. Mam2 is a pheromone receptor; Ste6 and Ppk33 are G0-activated guanyl nucleotide exchange factor (GEF) and protein kinase, respectively. These genes are under the control of the *S. pombe* Ste11 differentiation-promoting transcription factor, which contains the high mobility group (HMG) box [35,39,40]. 

Eight transcripts revealed an increase in ∆klf1 mutant cells relative to WT cells in G0. Their functions are diverse, but three of them (SPAC11D3.01c, SPAC869.09, SPCC70.08c) are strikingly responsive to oxidative stress. Another three (SPBC2G2.17c, SPCC74.04, Ctr4) are located next to the cell wall or plasma membrane. Klf1 might be involved in stress responses and cell surface homeostasis. The transcript level of an enolase, Eno102, involved in sugar catabolism was increased in WT cells in G0, but further increased in ∆klf1 cells in G0. The spbc2G2.17c^+^ gene product (similar to *S. japonicas* Psu2) is presumed to be the cell wall-localized beta-glucosidase involved in biogenesis of the cell wall (PomBase). Its transcript level greatly increased in WT in G0, and further increased in ∆klf1 cells in G0. Aes1 is an enhancer of antisense RNA-mediated gene silencing [41], and this transcript was decreased in WT cells in G0, but not in ∆klf1 cells. Why this transcript was not decreased in ∆klf1 G0 relative to WT G0 is unknown.

As the level of enolase Eno101 increased in G0, we suspected that glucose catabolism might be enhanced in ∆klf1 mutant cells. Nearly all mitochondrial genome transcripts (SPMIT.01~SPMIT.11) were activated approximately 2-fold in the G0 phase, while the transcripts for glucose transporters did not increase (Figure S3). Transcripts levels of aerobic respiration (red) and ribosome biogenesis (yellow) were mostly unchanged from wild-type levels, so a systematic increase of mitochondrial gene expression in ∆klf1 cells in G0 might be significant (Figure S4).

### Analysis of metabolites in ∆klf1 mutant G0 cells

To understand metabolic states, we quantified intracellular metabolites in ∆klf1 mutants in G0 using a metabolomic analysis recently developed for *S. pombe* [26,27]. Metabolites were extracted from different cultures (WT and ∆klf1 in the VE and G0 phase for 1-21 d) and analyzed by LC/Orbitrap MS. Thousands of metabolite peaks were detected, but only a fraction (~200) of them were firmly identified with standard compounds and a few were predicted by tandem MS analysis and precise chemical formula assignment [29]. Metabolic patterns differed considerably between the VE and G0 phases, and will be described in detail elsewhere. Here we focus only on the difference between WT and ∆klf1 in G0.

**Figure 7:**
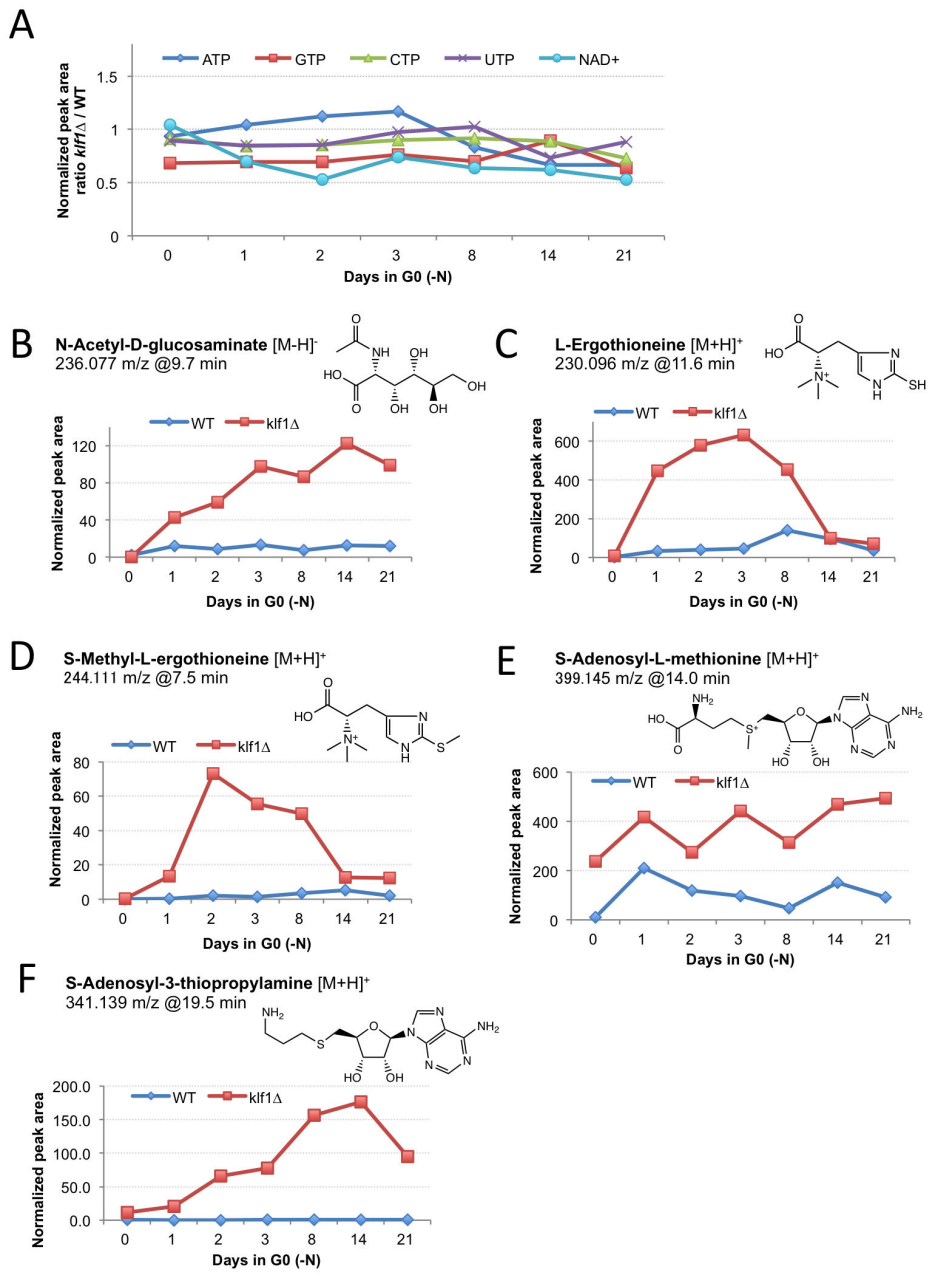
Metabolomic analysis of ∆klf1 mutant cells in G0 phase. The mass-to-charge (m/z) values and retention times (min) of each of the key metabolites are indicated together with their chemical structure. A. Levels of metabolites related to cellular energy are similar between wild-type and ∆klf1 mutants in G0 phase. B. N-acetyl-D-glucosaminate levels increased in ∆klf1 mutants (red) in G0 phase, while those in WT cells did not. C and D. Ergothioneine and S-methyl ergothioneine levels peaked in ∆klf1, but not in WT, 2 to 3 d after G0 phase. E and F. S-Adenosyl methionine (SAM) level was high throughout culture in both VE and G0. In contrast, the level of S-adenosyl-3-thiopropylamine (verified by its standard compound) levels peaked after 2 weeks in ∆klf1. Note that equal numbers of cells were used for the preparation of WT and ∆klf1 samples, therefore minor fluctuation of the metabolite peaks could be attributed to the difference in the average cell size. However, such fluctuation is negligible compared to the scale of the actual measured difference in metabolite levels.

We first examined levels of high-energy phosphate metabolites ATP, GTP, CTP, UTP, and NAD^+^ in ∆klf1 and WT cells. Levels were similar between WT and ∆klf1 in the G0 phase for 1 to 21 d (Figure 7A), suggesting that ∆klf1 mutant cells are metabolically active, despite their lost ability to restore cell division.

Several compounds, including N-acetyl-D-glucosaminate (NAG), showed a highly reproducible increase in ∆klf1 mutant G0 cells relative to WT G0 cells (Figure 7B). NAG levels were low in WT and ∆klf1 in VE, slightly increased in WT cells in G0, but strongly increased in ∆klf1 cells in G0. The role of NAG may be related to N-acetyl glucosamine, the chitin precursor. NAG was apparently not metabolizable, and accumulated.

Ergothioneine (EGT), an antioxidant compound derived from trimethylhistidine and cysteine [26], and its S-methyl derivative (Sm-EGT) greatly increased, although the peak appeared only transiently (Figure 7C, D). Levels of these compounds were low in both WT and ∆klf1 mutants in VE. Oxidative stress might be increased in ∆klf1 cells due to the increased activity of mitochondria. 

Levels of S-adenosyl methionine (SAM; Figure 7E) and its decarboxylated variant, S-adenosyl-3-thiopropylamine (Figure 7F), were greatly influenced in ∆klf1. SAM was already abundant (20-fold increase) in ∆klf1 VE cells. In G0, the difference became smaller (3-8 fold) as the level of SAM increased in the WT G0. The physiologic relevance of the increase of this methyl donor remains to be clarified.

## Discussion

The fission yeast zinc-finger transcription factor, Klf1, is essential to sustain long-term G0 quiescence [13]. In this study, we show that, in the absence of Klf1, a unique phenotype with the drastically aberrant cell morphology occurs in quiescent cells. To date, among thousands of mutants searched by cytologic screening, we have found no other mutant displaying the same phenotype [13,18; Sajiki et al., unpublished result]. ∆klf1 mutants often had a 10-fold larger cell volume after a four-week quiescent phase. Such an ‘obese’ cell phenotype might be considered a kind of senescence defect in non-dividing cells. Metabolomic data suggested that these quiescent mutant cells were still metabolically active, as the levels of nucleotide triphosphates were normal, as in WT, although the ability to restore cell division in the replenished medium was lost. We thus concluded that the long-term G0 phenotype of ∆klf1 uniquely disrupts the homeostasis of quiescence, allowing for an increase in cell volume, one of the features of growing cells.

The alteration of ∆klf1 cell wall morphology was subtle during the first week in G0 phase, became visible after two weeks, and dominant after four weeks. A small, but continuous and irreversible imbalance in anabolism or catabolism may occur in ∆klf1 G0 cells, eventually causing the fatal failure to exit G0 after medium replenishment. The cause of the volume increase appears to be the accumulation of chitin-like material, which may contain N-acetyl glucosaminate or its derivatives. Thin-section electron micrographs indicated that deposited materials formed layers or fibrous networks in the vicinity of the cell wall or the periplasmic space. The abnormal deposits appear to cause severe deformation of the cytoplasm and cell shape, and these features were irreversible. In addition, the frequency of peeled-off cell wall structures suggests that aberrant de novo cell wall assembly occurred in ∆klf1 G0 cells, possibly resulting in dissociation of the initial cell wall. The primary role of Klf1 might be to restrain imbalanced, production of cell wall precursor compounds. Klf1 may maintain the longevity of G0 phase cells, possibly through balanced cell wall metabolism. Klf1 might also restrain metabolism related to cell growth in G0 quiescence. Approximately 2% of ∆klf1 G0 cells are long and rod-shaped after two weeks in G0 phase. These elongated cells were never observed in WT G0 phase. We thus consider that the study of Klf1 may offer a unique opportunity to investigate homeostasis of cell volume or shape in the quiescent phase.

According to PomBase [23], *S. pombe* has 36 C2H2 zinc finger transcription factors, 11 of which (Ace2, Prz1, Clr1, Scr1, Rsv1, Klf1, Zas1, Rst2, SPAC25B8.19c, SPAC144.02, SPAC11D3.17) contain Krüppel-like C2H2 motifs that are spaced with 7 amino acids. Zas1 and Klf1 are similar (32% identical), and Rsv1 and Scr1 are also similar. The remaining seven are not significantly similar to each other except for the C2H2 domains. Zas1 is essential for viability [42]. The level of Klf1 in G0 is higher than that in vegetative growth. Results of transcriptome analysis of ∆klf1 G0 cells suggest that Klf1 directly or indirectly upregulates five genes (mam2^+^, ste6^+^, spk1^+^, rgs1^+^, ppk33^+^), the transcripts of which increase under N-starvation [12]. These genes belong to the group implicated in establishing sexual development and cell differentiation, which is known to be under the control of Ste11, an HMG-box transcription factor [35,38,43]. Three genes, spk1+, rgs1+, ppk33+, contain the sequence motif TTCTTTGTT in the promoter region, which perfectly matches the Ste11 recognition sequence [35], suggesting that these may be direct targets of Ste11. Another common sequence CCATTG was present in the promoter regions of all five genes. The relationship of the two transcription factors Ste11 and Klf1, however, remains to be studied. While their target genes may overlap, their functions appear to be distinct. Klf1 is essential for G0 maintenance, but it is not required at all for meiosis or sporulation. Ste11 is required for mating and sexual development [35].

Results of transcriptome analysis also suggest that Klf1 might downregulate eight functionally diverse genes that are implicated in cell wall renewal, oxidative stress response, glycolysis, nutrient uptake, RNA-mediated chromatin silencing, glycosidation, and methylation. Three of the genes (spac869.09^+^, spac11D3.01c^+^, spcc74.04^+^) strongly respond to oxidative stress [12]. Three others (spbc2g2.17c^+^, spcc74.04^+^, ctr4^+^) are located at the cell envelope or plasma membrane for wall import or renewal. Taken together, Klf1 may provide homeostatic control of G0 phase through tuning these positive and negative elements of cell differentiation and growth. Failure on either side of the regulation may cause a G0 phase imbalance.

Higher eukaryotic Krüppel factors are implicated in cell proliferation, differentiation, growth, development, survival, responses to external stresses, and cancer [44,45,46,47,48]. KLFs are involved in development and homeostasis of many organ systems [49,50]. We present evidence that Klf1 and Zas1 are associated in G0 phase. Drosophila Krüppel factors form heterodimers and act as transcriptional repressors [45]. As Klf1 is essential for G0, and Zas1 is not, the heterologous complex may be involved in G0 phase maintenance. Interestingly, while zas1^+^ encodes a protein with three zinc fingers, it is expressed predominantly to produce a protein containing only two zinc fingers. A third zinc finger resides within the in-frame intron that is normally spliced out. By reverse transcription-polymerase chain reaction analysis, Okazaki and Niwa [42] detected a minor transcript that encodes a protein with three zinc fingers, which is novel for lower eukaryotes. Such presumed alternative splicing for an assortment of zinc finger domains has been reported in animals and is implicated in switching of target genes expressed specifically during development. Note that Klf1 has two zinc fingers. An attractive possibility is that Zas1’s splicing to increase the two C2H2-type zinc finger domain to a three C2H2-type zinc finger domain might be related to G0 phase control. The resulting heterologous complex might be endowed with the ability to induce G0 quiescence. Further studies are clearly needed to clarify the interaction between Klf1 and Zas1.

In summary, this study shows that Klf1 has a unique function to maintain cell size and cell morphology in G0 quiescence. It is implicated in chronologic cell longevity, homeostasis of non-dividing cells, cell wall maintenance, and oxidative stress response. Klf1 is an excellent model for understanding metabolic factors that govern cellular quiescence.

## Supporting Information

Figure S1 **A**. ***S. pombe* wild-type cells grown vegetatively (VE) or arrested in G0 phase for 24 h were observed after staining with DAPI**.
Auto-fluorescence was negligible in the green channel, indicating that GFP images shown are not due to auto-fluorescence. B. Immunoblot analysis was performed to detect Klf1-GFP protein in VE and G0 cells. For control, extracts of wild-type (WT) cells not containing the chromosomally integrated klf1-GFP were used. The loading control was alpha-tubulin detected by antibody TAT1 (Materials and Methods). The level of Klf1-GFP in G0 cells was higher than in VE cells, as represented by Klf1-FLAG expression (Fig 1C).
(TIF)

Figure S2 **Wild-type and ∆klf1 mutant cells in vegetative (VE) and G0 states for 1 and 14 d were observed after staining with calcofluor, which binds to chitin.** In mutant cells, intense staining of calcofluor was observed (see text).
(TIF)

Figure S3 **Most transcripts of *S. pombe* SPMIT genes , which are encoded by the mitochondrial genome, were ~2-fold upregulated in ∆klf1 G0 mutants, whereas most transcripts of glucose transporters (Ght1-8) were similar (< 2-fold) between wild-type and ∆klf1 mutant cells in G0.**
(TIF)

Figure S4 **Transcriptomic comparison by scatter plot between ∆klf1 and wild-type of genes encoding aerobic respiration (red) and ribosome biogenesis (yellow).** No significant differences were observed.
(TIF)

Table S1 **Cell size distribution during long-term incubation under N-starvation.**
(XLSX)

Table S2 **Transcriptomic data from DNA microarray analysis of *∆klf1* mutant and wild type cells in G0 (1 day) and in VE.** Results of two independent experiments are shown.
(XLSX)
