# Supplementary figures and images for: Klf1, a C2H2 Zinc Finger-Transcription Factor, Is Required for Cell Wall Maintenance during Long-Term Quiescence in Differentiated G0 Phase
Source: PLoS One. 2013 Oct 22;8(10):e78545. doi: 10.1371/journal.pone.0078545 (PMC3805531; doi:10.1371/journal.pone.0078545)

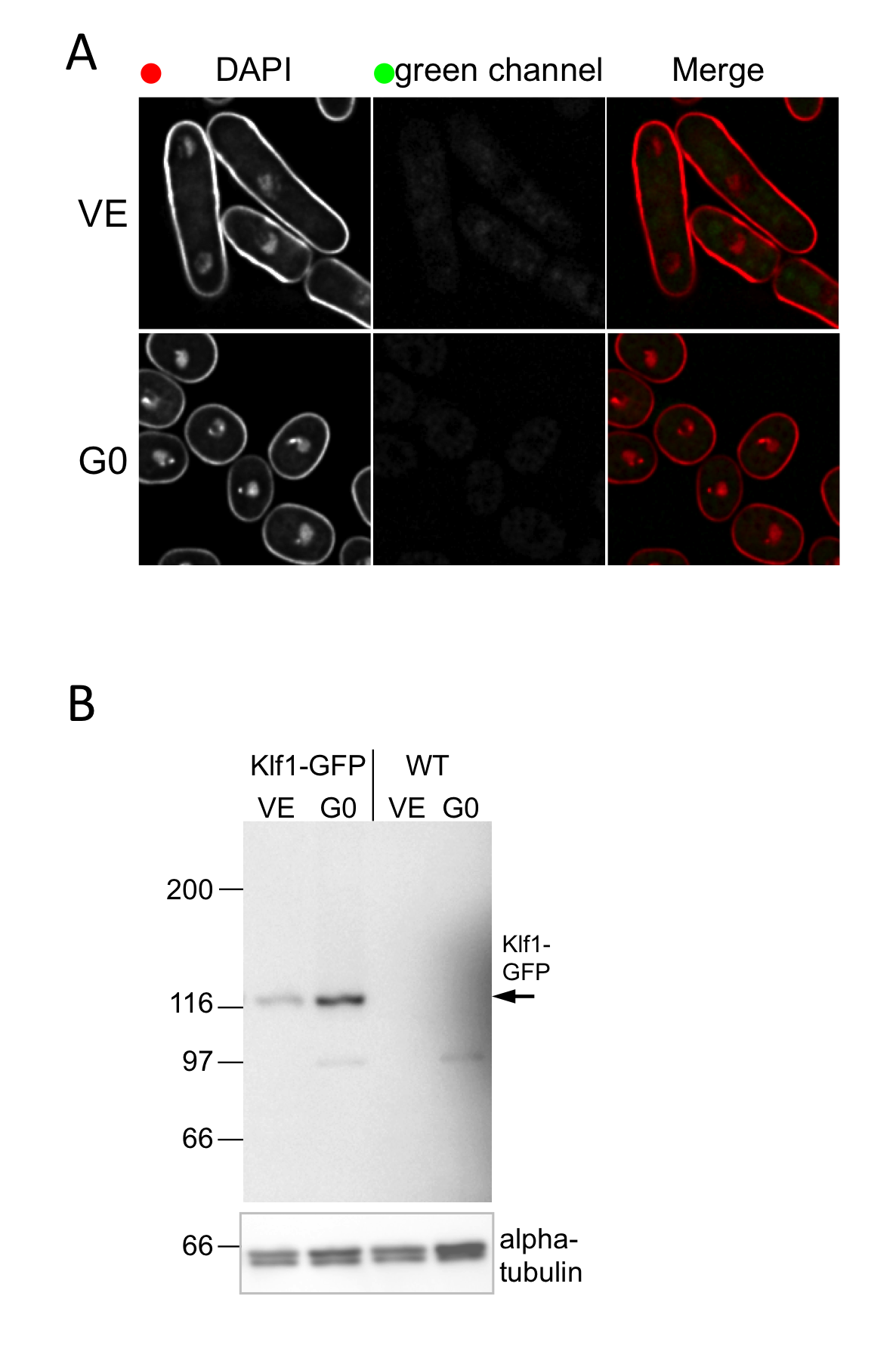

Supplement: Figure S1 — A. S. pombe wild-type cells grown vegetatively (VE) or arrested in G0 phase for 24 h were observed after staining with DAPI. Auto-fluorescence was negligible in the green channel, indicating that GFP images shown are not due to auto-fluorescence. B. Immunoblot analysis was performed to detect Klf1-GFP protein in VE and G0 cells. For control, extracts of wild-type (WT) cells not containing the chromosomally integrated klf1-GFP were used. The loading control was alpha-tubulin detected by antibody TAT1 (Materials and Methods). The level of Klf1-GFP in G0 cells was higher than in VE cells, as represented by Klf1-FLAG expression (Fig 1C). (TIF) [file pone.0078545.s001.tif]

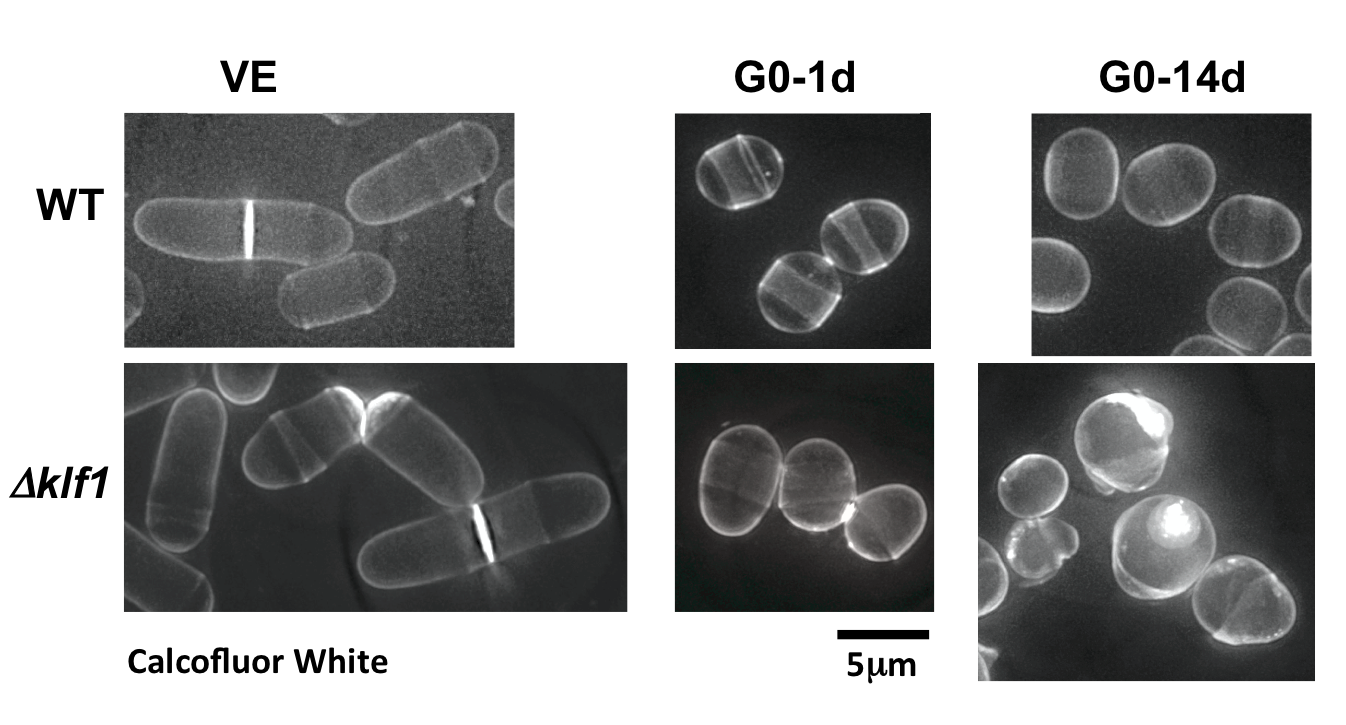

Supplement: Figure S2 — Wild-type and ∆klf1 mutant cells in vegetative (VE) and G0 states for 1 and 14 d were observed after staining with calcofluor, which binds to chitin. In mutant cells, intense staining of calcofluor was observed (see text). (TIF) [file pone.0078545.s002.tif]

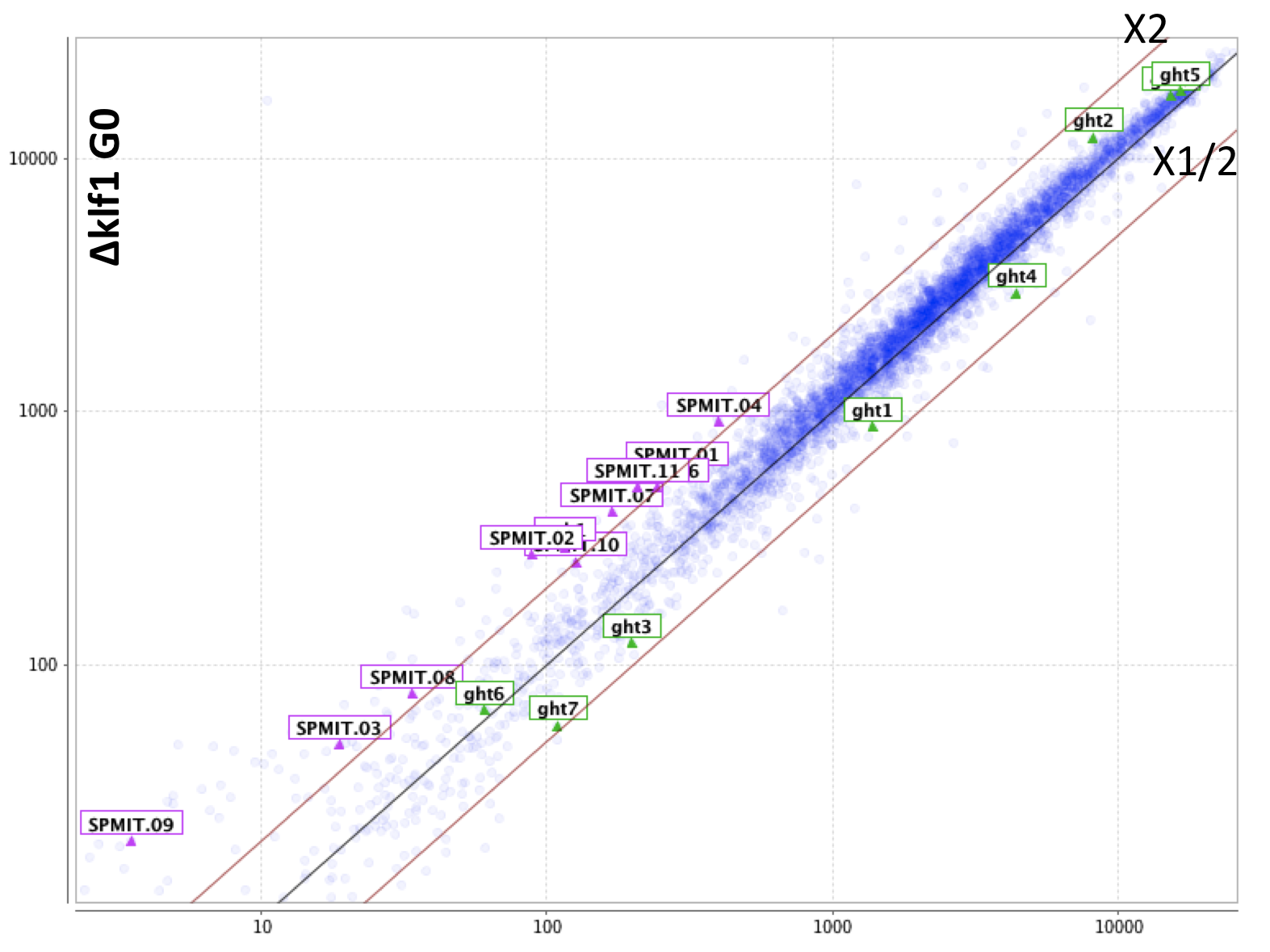

Supplement: Figure S3 — Most transcripts of S. pombe SPMIT genes , which are encoded by the mitochondrial genome, were ~2-fold upregulated in ∆klf1 G0 mutants, whereas most transcripts of glucose transporters (Ght1-8) were similar (< 2-fold) between wild-type and ∆klf1 mutant cells in G0. (TIF) [file pone.0078545.s003.tif]

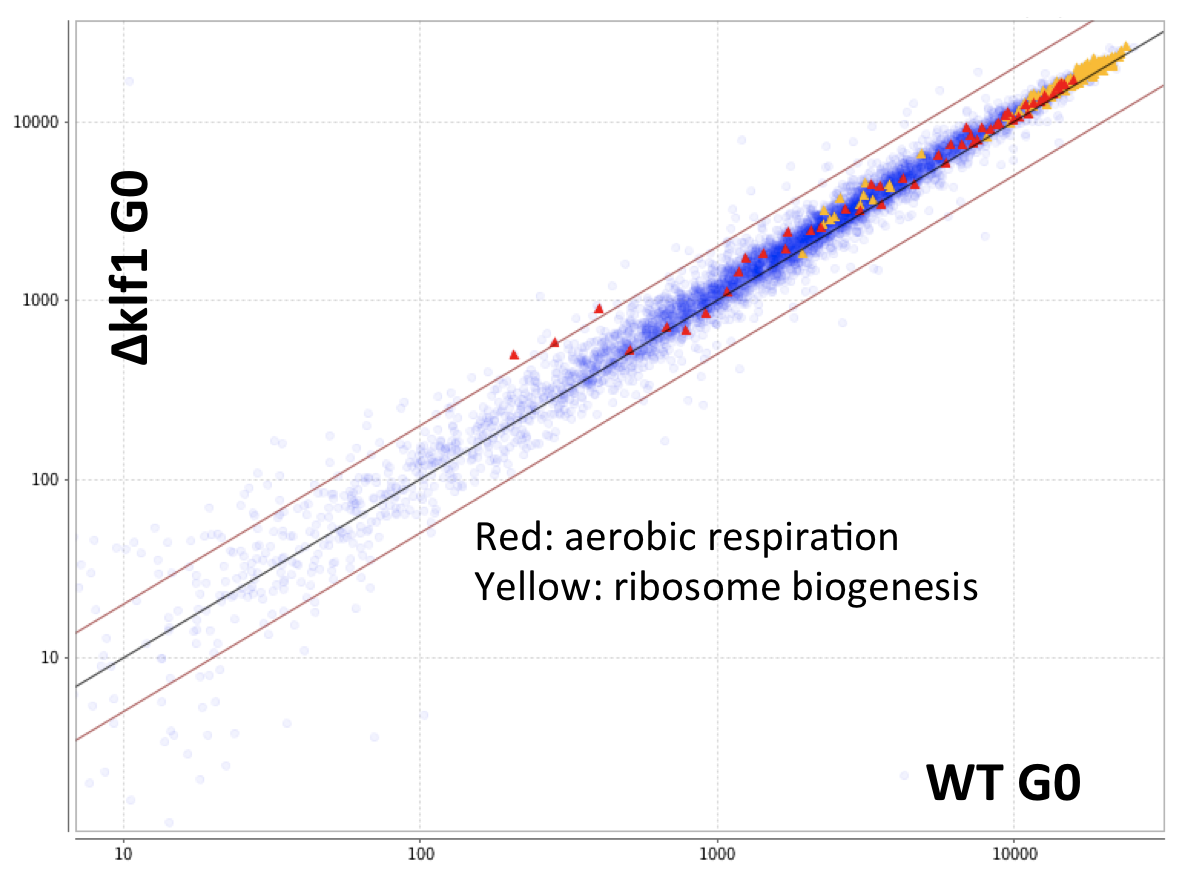

Supplement: Figure S4 — Transcriptomic comparison by scatter plot between ∆klf1 and wild-type of genes encoding aerobic respiration (red) and ribosome biogenesis (yellow). No significant differences were observed. (TIF) [file pone.0078545.s004.tif]
